# MicroRNA-16 feedback loop with p53 and Wip1 can regulate cell fate determination between apoptosis and senescence in DNA damage response

**DOI:** 10.1371/journal.pone.0185794

**Published:** 2017-10-02

**Authors:** Maria Vitória C. Issler, José Carlos M. Mombach

**Affiliations:** Department of Physics, Universidade Federal de Santa Maria, Santa Maria, Rio Grande do Sul, Brazil; Cornell University, UNITED STATES

## Abstract

Cell fate regulation is an open problem whose comprehension impacts several areas of the biosciences. DNA damage induces cell cycle checkpoints that activate the p53 pathway to regulate cell fate mechanisms such as apoptosis or senescence. Experiments with different cell types show that the p53 pathway regulates cell fate through a switch behavior in its dynamics. For low DNA damage the pathway presents an oscillatory pattern associated with intense DNA damage repair while for high damage there are no oscillations and either p53 concentration increases inducing apoptosis or the cell enters a senescence state. Apoptosis and senescence phenotypes seem to have compensatory functions in tissues and the microRNA 16–1 (miR-16) is involved in the regulation of the fate between both phenotypes in cancer cells. To investigate the regulation of cell fate we developed a logical model of the G1/S checkpoint in DNA damage response that takes into account different levels of damage and contemplates the influence of miR-16 through its positive feedback loop formed with p53 and Wip1. The model reproduces the observed cellular phenotypes in experiments: oscillatory (for low DNA damage) regulated by negative feedback loops involving mainly p53 and Mdm2 and apoptotic or senescent (for high DNA damage) regulated by the positive p53/Wip1/miR-16 feedback loop. We find good agreement between the level of DNA damage and the probability of the phenotype produced according to experiments. We also find that this positive feedback makes senescent and apoptotic phenotypes to be determined stochastically (bistable), however controlling the expression level of miR-16 allows the control of fate determination as observed experimentally.

## Introduction

DNA damage is a threat to genome integrity and its protection relies on the tumor protein, p53, signaling pathway response to the threat. The activity of the p53 pathway involves several feedback loops that control phosphorylated p53 concentration levels and can influence in different ways the expression of gene sets that lead to specific cell fates [1–3]. In general, positive feedback loops are associated with cell fate stabilization and negative feedback loops with reversible cell fates [4, 5]. Under DNA damage the cell cycle is arrested at checkpoints activating the p53 pathway dynamics, in the case of light DNA damage an oscillatory dynamics is observed while for heavy damage, senescence (permanently cell cycle arrested cells) or apoptosis pathways are triggered [2, 6]. Apoptosis and senescence phenotypes seem to have compensatory functions, however the molecular mechanisms that regulate senescence are yet unclear and of utmost importance to study aging [7–9]. Experimental and theoretical attempts to describe the oscillatory and apoptotic phenotypes are in progress, but in the case of senescence more investigations are required. Recently, an experiment confirmed a correlation between the DNA damage level induced by the anti-cancer drug etoposide with a switch in the p53 pathway behavior. For low concentrations of the drug culture cells present an oscillatory phenotype and few cell deaths, while for high concentrations there are arrested cells, no oscillations, and many cell deaths [2, 3, 10]. The onset of senescence is associated mainly with the upregulation of the cell cycle inhibitors pRB (retinoblastoma 1 protein), p21 (cyclin-dependent kinase inhibitor 1A), and/or the senescence DNA locus CDKN2A (cyclin-dependent kinase inhibitor 2A) [7]. MicroRNAs (miRNAs) can also regulate cell cycle. For example, microRNAs can form feedback loops with p53 [11]. MiRNAs are small (20–24 nt) noncoding regulatory RNA molecules that target specific mRNAs to repress their translation. A recent experimental study by Kitadate and coworkers confirmed that microRNA 16–1 (miR-16), whose expression is regulated by p53, mediates the fate between senescence or apoptosis through p21 in cutaneous T-cell and other non-Hodgkin lymphomas. Their result supports the hypothesis that both phenotypes have important compensatory functions in tissues [8, 12, 13]. By changing miR-16 expression level the authors observed a phenotype change from senescence to apoptosis in cells. These experimental observations provide a basis for understanding how the p53 pathway dynamics is determined by repairable or irreparable DNA damage.

Aiming to describe more realistically the mechanisms of cell fate regulation, in this work we model the regulation of the p53 pathway under DNA damage and show that the feedback loop formed by miR-16, p53, and its inhibitor, Wip1 (protein phosphatase Mg2+/Mn2+ dependent 1D), affect cell fate determination. In order to approach this problem we introduce a logical model of the G1/S cell cycle checkpoint regulation. Logical modeling is receiving increasing interest in regulatory networks research (for reviews see [14, 15]). Among many applications, logical modeling was applied to cell fate regulation in cancer [16], to study drug synergies [17], and cellular senescence [18, 19].

### The G1/S checkpoint molecular mechanisms

In what follows we define our logical model based on the molecular interactions involved in the activation by DNA damage of the G1/S cell cycle checkpoint that is p53-dependent (for a review see [20, 21]). Our basic hypothesis in this model is that cell fate determination happens at checkpoints.

Mdm2 (MDM2 proto-oncogene) is the main regulator of p53. In the absence of DNA damage unphosphorylated p53 concentration remains low by degradation due to MDM2 mediated p53 ubiquitination [22]. Upon DNA damage the cell cycle checkpoint pathways G2/M and G1/S are activated [20, 21]. Here, for simplicity, we focus only on the G1/S checkpoint and p53-p21 regulated senescence. CDKN2A can also contribute to senescence, however for cell fate regulation involving miR-16 (as reported in the work of Kitadate et al.) it does not seem to play a major role and we will not consider it in the present model [12].

**Fig 1:**
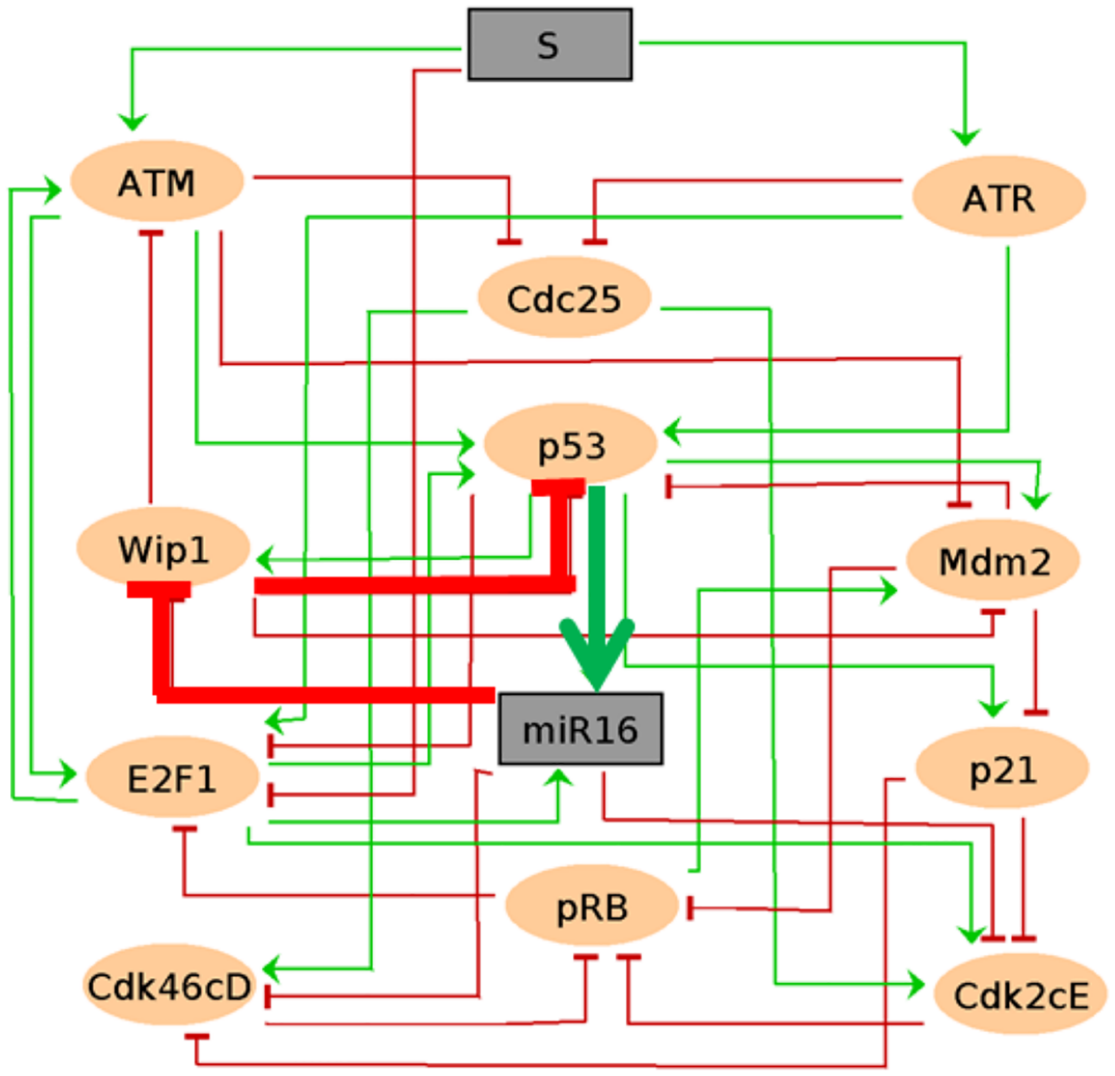
Regulatory network for the G1/S checkpoint pathway. Elliptic nodes represent proteins and rectangular nodes represent inputs or microRNA. The input node in grey at the top of the network denotes DNA damage level. Green lines represent activations and red lines inhibitions.

The G1/S checkpoint initiates through p53 phosphorylation by ATM (ataxia-telangiectasia mutated) and ATR (ataxia telangiectasia and Rad3 related) kinases that are activated by upstream DNA damage response (DDR) molecules [20, 21]. ATM and ATR pathways are not completely independent. There is a considerable amount of pathways crosstalk (reviewed in Gobbini [23]) in the activation of the checkpoint. Phosphorylated p53 is more resistant to Mdm2 mediated p53 ubiquitination, altering its time balance with this protein. p53 is also required for Mdm2 expression, making them a negative circuit [24]. The network can be seen in Fig 1 and a description of the main interactions is given in what follows. Detailed bibliographical information of each node interaction can be found in the supporting S1 File and the GINsim version of the model in the supporting S2 File.

Upon DNA damage the ATM and ATR pathways initiate a cascade of phosphorylations that activate p53 and inhibit Cdc25A (Cell division cycle 25A protein) [21]. ATM is activated by DNA double-strand breaks (DSBs), while ATR by DNA single-strand breaks (SSBs). ATM also phosphorylates Mdm2, preventing it from targeting p53 for degradation. p21 is activated by p53 and its degradation is promoted by Mdm2 [25]. Cdc25A is a phosphatase required to activate the protein complexes, cyclin-dependent kinase 4 and 6-Cyclin D (Cdk46cD) and cyclin-dependent kinase 2-cyclin E (Cdk2cE), which promote the cell cycle phase transition. Activated Cdk46cD phosphorylates the retinoblastoma 1 protein (pRB) and promotes the release of E2F1 (transcription factor 1) that is required in the S phase [20, 21]. MiR-16 targets the mRNA of Wip1 and Cyclins E and D, negatively regulating the expression of these proteins [13]. Wip1 is activated by p53 [26] and can deactivate by dephosphorylation Mdm2, p53, and ATM [26, 27]. The positive circuit involving p53, miR-16, and Wip1 (see Fig 1) is described as follows: p53 is required for Wip1 upregulation which can dephosphorylate Mdm2 affecting its interaction with p53 [28]. p53 enhances the post-transcriptional maturation of miR-16 [29] that targets the mRNA of Wip1, downregulating it [30]. Alternatively, Wip1 can dephosphorylate p53 decreasing its activation [31]. The molecular description of the model elements is presented in the supporting S1 Table.

## Materials and methods

### Logical modeling

Here we briefly describe the logical formalism. For a deeper description see ref. [15].

A logical model of a regulatory network (*G*, *K*) is defined by a set of *n* discrete regulatory components, *G* = (*g*_1_, *g*_2_, …*g*_*n*_), where each *g*_*i*_ takes its values in (0, …*M*_*i*_), representing the range of functional levels of the component. The components can represent molecule concentrations or biological states, biological processes (e.g. a pathway), or phenotypes (apoptosis, senescence etc.) A multivalued *g*_*i*_ is considered when the variable has different effects on a target or acts at different levels on distinct targets. Input components are not regulated and represent constant external conditions of the environment.

The state space S of a logical model is finite and a state is a vector *g* = [*g*_1_, *g*_2_, …*g*_*n*_]. A logical function *K*_*i*_ defines the values of each *g*_*i*_ in terms of activatory or inhibitory edges connecting the *g*_*i*_, which characterizes a directed graph. The transition function *K* is defined as *K*(*g*) = (*K*_1_(*g*), …*K*_*n*_(*g*)). multivalued variables are increased or decreased by 1 stepwise.

State transition graphs (STG) are used to describe the asymptotic behaviors in the dynamics of logical models which are called attractors. End nodes which have no successor state in STG, i.e. *K*(*g*) = *g*, correspond to a stable state or, in contrast, the trajectory in the STG can have a cyclic attractor. The most common update schemes of a logical model are the synchronous and asynchronous methods. The synchronous method generates a completely deterministic dynamics by updating all variables at each time step, while in the asynchronous each variable is updated independently and can generate stochastic behavior. Here we use the more general asynchronous update defined as follows for all *i* ≠ *j*:

$$\begin{array}{c} g_{i}\left(t+1\right)=g_{i}\left(t\right)+Sign\left(K_{i}\left(g\left(t\right)\right)-g_{i}\left(t\right)\right),\;g_{j}\left(t+1\right)=g_{j}\left(t\right). \end{array}$$ (1)

The logical method allows simulation of perturbations, known experimentally as loss of function (LoF) or gain of function experiments (GoF), which consist in fixing a variable to its lowest levels or to its highest levels respectively. The method also permits consideration of different time scales in the same model as it happens with transcriptional regulation and protein phosphorylation processes. Regulatory circuit analysis allows identifying circuits that play a role in the emergence of dynamical properties; negative feedbacks (referred here as negative circuits) encompass an odd number of inhibitions and are required for oscillations (determined by a complex attractor in the dynamics), whereas positive feedbacks (here referred as positive circuits) encompass an even number of inhibitions and are required for multi-stability [15].

In this work we used the tool GINsim 2.9.5 (download from: http://compbio.igc.gulbenkian.pt/nmd/node/82) [15] which implements several algorithms for analysis of logical models including determination of stable states, perturbations and determination of attractors reachability [32]. The code used in the simulations is available from the supporting S2 File.

### Input, multivalued nodes, attractors and phenotypes

In order to contemplate different DNA damage levels, we define the input S (damage) of the network as multivaluated, having three states representing: no damage (=0), low damage (=1), and high or irreparable damage (=2). Consequently, the nodes directly regulated by S, ATM, and ATR are affected proportionally to the input level S and we define them also as multivaluated. All other nodes with exception of p53, E2F1, and miR16 are defined as boolean for simplicity. p53 expression pattern is complex. As observed experimentally, its levels can induce different cell fates [3, 33]. It is inhibited in the absence of DNA damage, induced in its presence, and when its concentration is high leads to cell apoptosis. So, we define p53 as having three states to mimic this 3 state pattern that is more realistic than a boolean case. Then in our model a stable state with p53 at its highest level 2 implies an apoptotic phenotype. E2F1 and miR16 are also involved in the regulation of different cell fates, so we define them as multivaluated to be more realistic, similar to p53.

## Results

**Fig 2:**
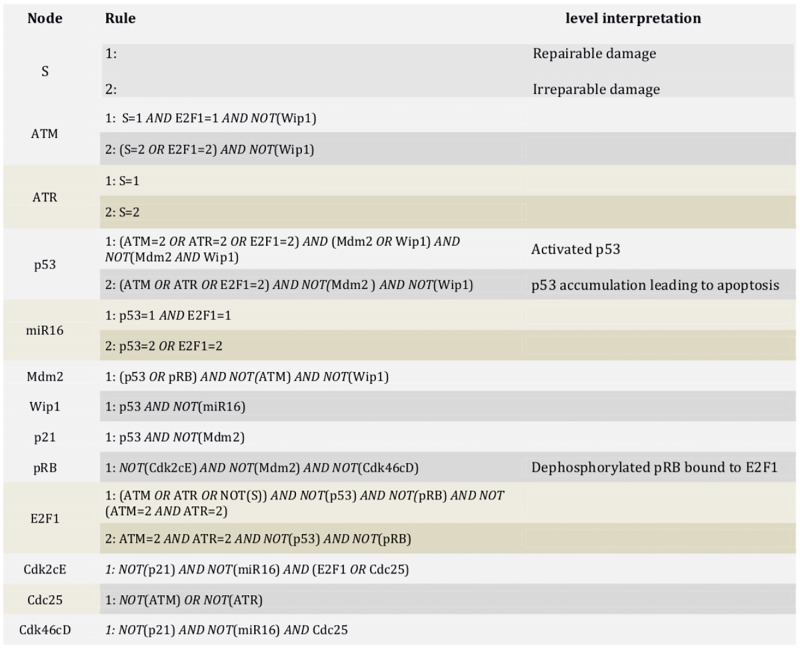
Logical rules. Rules controlling the states of the network nodes based on the biochemical literature (see S1 File). 0 is the default value.

In our results, we associate stable phenotypes with stable states and cyclic attractors with transient phenotypes [4, 15]. Then, based on experimental evidence, the interpretation of phenotypes produced by the model is as follows: a proliferative state corresponds to a stable state where the G1/S phase transition promoters (E2F1, Cdk2cE, Cdk46cD) are activated, implying that the cell cycle was not arrested at the G1 phase. There are two types of cycle arrests: stable and transient ones. Stable arrests correspond to stable states where the phase transition promoters are inhibited, and transient arrests correspond to a cyclic attractor. Senescence corresponds to a stable arrest where in addition both pRB and p21 are activated [7, 8]. The rules controlling each node are presented in Fig 2.

### Stable states of the wild-type case

**Fig 3:**
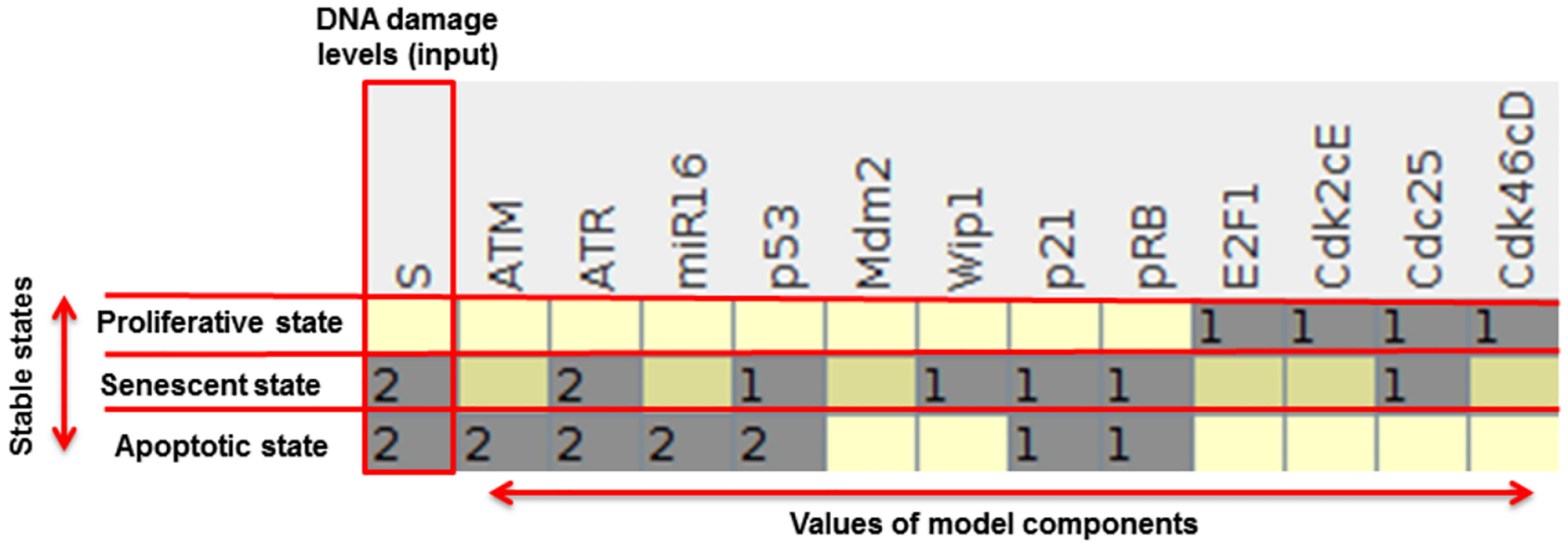
Stable states of the wild-type case. The left-most column lists the DNA damage levels that lead to stable states. Each line is a unique stable state characterized by the value of the components and the corresponding phenotype is indicated (see section Material and methods). Numbers stand for variable values and empty spaces correspond to value zero.

In Fig 3 we present the 3 stable states of the model for the wild-type case. For an initial state where all variables are initially null, the input S = 0 produces a stable state corresponding to proliferation.

For S = 1 there is no stable state. Instead the system is trapped in a cyclic attractor with 4608 states where all nodes oscillate but ATR. We associate this phenotype to the oscillatory behavior observed experimentally for low DNA damage. This result shows that the model reproduces the switch in dynamics (see below), i.e. the change from oscillatory to non-oscillatory behavior determined by the DNA damage level [2]. Among the many circuits of the network the only functional negative circuits determined by GINSim for (S = 1) are: p53/Mdm2, p53/Wip1, pRB/Mdm2, ATM/Cdc25/Cdk46cD/pRB/E2F1. So, the cyclic behavior results from the interference of these negative circuits. Besides p53, oscillations of other proteins belonging to the G1/S checkpoint were already observed experimentally for ATM, Wip1, p21, Mdm2, miR-16, pRB, E2F1, Cdc25, Cdk2, Cdk4 [3, 10, 34].

For S = 2, there are no oscillations and the model predicts a bistability between two different arrest states, in one case p53 = 2, which we defined as an apoptotic state, and in the other case the state corresponds to senescence [35]. This result suggests that senescence and apoptosis should coexist in DNA damage experiments, which is indeed observed [6]. There is a consensus that irreparable DNA damage produces apoptotic and senescent cells, so it is reasonable to assume that these two phenotypes may constitute a bistable state which can address in part their compensatory functions [8, 12]. In agreement with experiments, Mdm2 is inhibited at high levels of damage [2] and we identify two functional positive circuits: p53/miR16/Wip1 and E2F1/Cdk2cE/pRB. In the Discussion section we show that only the first circuit controls the dynamics for S = 2.

Summarizing, in the absence of DNA damage the only solution is proliferation and for low damage, the negative circuits dominate, generating the cyclic dynamics. For high damage there are no oscillations and the positive circuit Wip1/p53/miR16 dominates the cell fate process.

### Wild-type case phenotype probabilities

In Table 1 we present an estimate of the probabilities of each phenotype for the wild-type case using the Avatar algorithm (exact exit probabilities) in GINsim with 750 runs. The other parameters of the algorithm were set to the default values. The algorithm performs an exhaustive search in the state space of the model to determine the size of the basin of attraction of each attractor [32]. The initial state of each node in the search was set to zero with the exception of the input S.

**Table 1:** Phenotype probabilities for the wild-type case and damage S.

phenotype	probability
proliferation	0.37(S = 0)
apoptosis	0.11(S = 2)
senescence	0.18(S = 2)
transient arrest	0.34(S = 1)

Probabilities of each phenotype for the wild-type case estimated with the Avatar algorithm (exact exit probabilities) in GINsim with 750 runs.

Below we present perturbations results of the main regulators of the system dynamics (Mdm2, miR16 and Wip1) whose influence is fully discussed in the next section.

### Mdm2 perturbations

Table 2 presents the estimated probabilities of each phenotype obtained *in silico* for node Mdm2 knockdown (KD), activation (O), and corresponding DNA damage (S): calculated with 500 runs of the Avatar algorithm. For S = 2 and Mdm2 KD there is bistability between apoptosis and senescence.

**Table 2:** Phenotype probabilities for KD and activation (O) of node Mdm2 according to DNA damage S.

phenotype	KD			O		
S	0	1	2	0	1	2
proliferation	1.0	0.0	0.0	0.0	0.0	0.0
apoptosis	0.0	1.0	0.45	0.0	0.0	0.0
senescence	0.0	0.0	0.55	0.0	0.0	1.0
transient arrest	0.0	0.0	0.0	1.0	1.0	0.0

For S = 2 and Mdm2 KD there is bistability between apoptosis and senescence.

### Mir16 perturbations

In Table 3 we present the *in silico* results for node miR16 KD, activation, and different levels of DNA damage S. We observe that for the same level of damage different miR16 expression levels cause a change in phenotypes.

**Table 3:** Phenotype probabilities for different levels of expression and DNA damage S for node miR16.

phenotype	KD			O1			O2		
S	0	1	2	0	1	2	0	1	2
proliferation	1.0	0.0	0.0	0.0	0.0	0.0	0.0	0.0	0.0
apoptosis	0.0	0.0	0.0	0.0	0.0	1.0	0.0	0.0	1.0
senescence	0.0	0.0	1.0	0.0	0.0	0.0	0.0	0.0	0.0
trans. arrest	0.0	1.0	0.0	1.0	1.0	0.0	1.0	1.0	0.0

KD: knockdown; O1: median expression (miR16 = 1). O2: overexpression (miR16 = 2).

### Wip1 perturbations

In Table 4 we present the *in silico* results for node Wip1 KD, activation, and different levels of DNA damage S. We find that any Wip1 perturbation destroys the bistable dynamics.

**Table 4:** Phenotype probabilities for KD and activation (O) of node Wip1 according to DNA damage S.

phenotype	KD			O		
S	0	1	2	0	1	2
proliferation	1.0	0.0	0.0	1.0	1.0	0.0
apoptosis	0.0	0.0	1.0	0.0	0.0	0.0
senescence	0.0	0.0	0.0	0.0	0.0	1.0
transient arrest	0.0	1.0	0.0	0.0	0.0	0.0

In what follows we discuss the model phenotype predictions when confronted with single node GoF and LoF perturbations and the experimental data available. We give special emphasis on the switch and bistable dynamics and their regulators. The results of all perturbations are listed in the supporting S2 Table.

## Discussion

The model presents agreement with experiments testing the influence of Mdm2 expression levels on the switch dynamics [2]. Accordingly, Mdm2 is the main regulator of the switch. In U-2 OS cells a low level of DNA damage using the chemotherapeutic drug etoposide induces most cells to an oscillatory phenotype with probability p≃0.8 and 0.2 for the other phenotypes [2]. While our model predicts p = 1 an oscillatory phenotype. When in addition to low damage Mdm2 is knockdown (KD), cells present an apoptotic phenotype with probability p≃0.8 and 0.2 for the other phenotypes. While our model predicts an apoptotic phenotype with probability 1. The model shows that the knockdown of Mdm2 or p53 nodes abrogate the oscillatory phenotype (see S2 Table). To verify the role of all negative circuits found for *S* = 1 we performed a complete knockout of all the elements of each negative circuit (p53/Mdm2, p53/Wip1, pRB/Mdm2, ATM/Cdc25/Cdk46cD/pRB/E2F1) and observed that only the knocked out circuits containing p53 or Mdm2 abrogated the cyclic dynamics. So, our results present fair agreement with the work of Chen et al. and we conclude that after p53, Mdm2 is the main regulator of the oscillatory dynamics, implying that the negative circuit ATM/Cdc25/Cdk46cD/pRB/E2F1 does not play an essential role in generating cyclic behavior. We also verified that this dynamic is robust against other LoFs and GoFs of the model elements including those of node miR16.

In Table 3 we investigated if the differential expression of node miR16 can change the fate between apoptosis and senescence, since according to the work of Kitadate et al., it mediates the regulation of these two fates in cutaneous T-cell and other non-Hodgkin lymphomas [12]. In our model the resulting phenotype depends on S, something that was not studied by the Kitadate et al. work. We observe that perturbations destroy the bistable dynamics, pushing the system to decay in a specific phenotype that can be changed according to miR16 expression level and DNA damage. For a fixed S, in the absence of DNA damage the model predicts a phenotype change from a proliferative one, when miR16 KD, to a transient arrest one, when miR16 = 1 or 2. For S = 1 the model predicts only transient arrest, while for S = 2 it presents a phenotype change from senescence to apoptosis with increasing miR16 expression level. This can be related to the findings by Kitadate et al. The induction of apoptosis by miR-16 overexpression was already observed in different cancer cells types [36, 37]. Additional perturbations of miR16 not included in Table 3 can be found in the supporting S2 Table.

Finally, we determined that the bistability is robust to several LoF and GoF perturbations of all network nodes showing that it is a general solution of our model and not a particular case. As we can see in Table 4 among the elements of the positive circuit p53/Wip/miR16, Wip1 is the main regulator since any of its perturbations destroy the bistable dynamics, while this is not true for the other two nodes (see also the S2 Table).

## Conclusion

In this work we proposed a model for cell fate regulation in DNA damage response involving the p53 pathway and miR-16. The model shows that under low damage the experimentally observed oscillatory phenotype is regulated by several negative circuits involving mainly p53 and Mdm2. For high damage the positive circuit Wip1/p53/miR16 dominates the dynamics making the fate determination between apoptosis and senescence bistable, however perturbations of miR-16 can destroy this mechanism allowing the control of cell fate.

## Supporting information

S1 File Molecular interactions. Bibliographical references of molecular interactions in the model.
(PDF)

S2 File GINsim code. The GINsim code used in this study. Requires GINsim 2.9.5 to process it, download from: http://compbio.igc.gulbenkian.pt/nmd/node/82.
(ZGINML)

S1 Table Molecules. Official name of molecules used in the model.
(PDF)

S2 Table Perturbations. List of GoF and LoF perturbations and corresponding phenotypes.
(PDF)
