# Supplementary material for: MicroRNA-16 feedback loop with p53 and Wip1 can regulate cell fate determination between apoptosis and senescence in DNA damage response
Source: PLoS One. 2017 Oct 2;12(10):e0185794. doi: 10.1371/journal.pone.0185794 (PMC5624635; doi:10.1371/journal.pone.0185794)
Supplement: S1 File — Bibliographical references of molecular interactions in the model. (PDF) [file pone.0185794.s001.pdf]

Bibliographical References of Molecular Interactions

- S $\rightarrow$ ATM [1, 2]
- S $\rightarrow$ ATR [1, 2]
- ATM $\dashv$ Cdc25 [3, 4] (*)
- ATR $\dashv$ Cdc25 [4]
- ATM $\rightarrow$ p53 [5, 6]
- ATM $\dashv$ Mdm2 [2, 7]
- ATM $\rightarrow$ E2F1 [8]
- ATR $\rightarrow$ E2F1 [8]
- ATR $\rightarrow$ p53 [9]
- Wip1 $\dashv$ p53 [10]
- Wip1 $\dashv$ Mdm2 [11]
- Wip1 $\dashv$ ATM [12]
- Mdm2 $\dashv$ p53 [13, 14, 15]
- Mdm2 $\dashv$ p21 [16]
- Mdm2 $\dashv$ pRB [17, 18]
- p53 $\rightarrow$ Mdm2 [19, 13, 15]
- p53 $\rightarrow$ p21 [20, 21]
- p53 $\rightarrow$ Wip1 [22]
- p53 $\rightarrow$ miR16 [23]
- E2F1 $\rightarrow$ p53 [24]
- E2F1 $\rightarrow$ ATM [25]
- E2F1 $\rightarrow$ miR16 [26]
- p21 $\dashv$ Cdk2cE [27]
- p21 $\dashv$ Cdk46cD [27]
- pRB $\dashv$ E2F1 [28]
- pRB $\rightarrow$ Mdm2 [29]

- miR16 ⊣ Cdk2cE [30]
- miR16 ⊣ Cdk46cD [30]
- miR16 ⊣ Wip1 [31]
- Cdc25 → Cdk2cE [32]
- Cdk2cE ⊣ pRB [33]
- Cdk46cD ⊣ pRB [34]

References

[1] Abraham RT. Cell cycle checkpoint signaling through the ATM and ATR kinases. Genes & Development. 2001;15(17):2177–2196. doi:10.1101/gad.914401.

[2] Khosravi R, Maya R, Gottlieb T, Oren M, Shiloh Y, Shkedy D. Rapid ATM-dependent phosphorylation of MDM2 precedes p53 accumulation in response to DNA damage. Proceedings of the National Academy of Sciences. 1999;96(26):14973–14977. doi:10.1073/pnas.96.26.14973.

[3] Falck J, Mailand N, Syljuasen RG, Bartek J, Lukas J. The ATM-Chk2-Cdc25A checkpoint pathway guards against radioresistant DNA synthesis. Nature. 2001;410(6830):842–847. doi:10.1038/35071124.

[4] Thanasoula M, Escandell JM, Suwaki N, Tarsounas M. ATM/ATR checkpoint activation downregulates CDC25C to prevent mitotic entry with uncapped telomeres. The EMBO Journal. 2012;31(16):3398–3410. doi:10.1038/emboj.2012.191.

[5] Banin S, Moyal L, Shieh SY, Taya Y, Anderson CW, Chessa L, et al. Enhanced Phosphorylation of p53 by ATM in Response to DNA Damage. Science. 1998;281(5383):1674–1677. doi:10.1126/science.281.5383.1674.

[6] Canman CE, Lim DS, Cimprich KA, Taya Y, Tamai K, Sakaguchi K, et al. Activation of the ATM Kinase by Ionizing Radiation and Phosphorylation of p53. Science. 1998;281(5383):1677–1679. doi:10.1126/science.281.5383.1677.

[7] Maya R, Balass M, Kim ST, Shkedy D, Leal JFM, Shifman O, et al. ATM-dependent phosphorylation of Mdm2 on serine 395: role in p53 activation by DNA damage. Genes & development. 2001;15(9):1067–1077.

[8] Lin WC, Lin FT, Nevins JR. Selective induction of E2F1 in response to DNA damage, mediated by ATM-dependent phosphorylation. Genes & Development. 2001;15(14):1833–1844.

[9] Tibbetts RS, Brumbaugh KM, Williams JM, Sarkaria JN, Cliby WA, Shieh SY, et al. A role for ATR in the DNA damage-induced phosphorylation of p53. Genes & Development. 1999;13(2):152–157.

[10] Takekawa M, Adachi M, Nakahata A, Nakayama I, Itoh F, Tsukuda H, et al. p53-inducible Wip1 phosphatase mediates a negative feedback regulation of p38 MAPK-p53 signaling in response to UV radiation. The EMBO Journal. 2000;19(23):6517–6526. doi:10.1093/emboj/19.23.6517.

[11] Lu X, Ma O, Nguyen TA, Jones SN, Oren M, Donehower LA. The Wip1 Phosphatase Acts as a Gatekeeper in the p53-Mdm2 Autoregulatory Loop. Cancer Cell. 2007;12(4):342–354. doi:https://doi.org/10.1016/j.ccr.2007.08.033.

[12] Shreeram S, Demidov ON, Hee WK, Yamaguchi H, Onishi N, Kek C, et al. Wip1 Phosphatase Modulates ATM-Dependent Signaling Pathways. Molecular Cell. 2006;23(5):757–764. doi:https://doi.org/10.1016/j.molcel.2006.07.010.

[13] Freedman DA, Wu L, Levine AJ. Functions of the MDM2 oncoprotein. Cellular and Molecular Life Sciences CMLS. 1999;55(1):96–107. doi:10.1007/s000180050273.

[14] Haupt Y, Maya R, Kazaz A, Oren M. Mdm2 promotes the rapid degradation of p53. Nature. 1997;387(6630). doi:http://dx.doi.org/10.1038/387296a0.

[15] Wu X, Bayle JH, Olson D, Levine AJ. The p53-mdm-2 autoregulatory feedback loop. Genes & Development. 1993;7(7a):1126–1132. doi:10.1101/gad.7.7a.1126.

[16] Zhang Z, Wang H, Li M, Agrawal S, Chen X, Zhang R. MDM2 Is a Negative Regulator of p21WAF1/CIP1, Independent of p53. Journal of Biological Chemistry. 2004;279(16):16000–16006. doi:10.1074/jbc.M312264200.

[17] Uchida C, Miwa S, Kitagawa K, Hattori T, Isobe T, Otani S, et al. Enhanced Mdm2 activity inhibits pRB function via ubiquitin-dependent degradation. The EMBO Journal. 2004;24(1):160–169. doi:10.1038/sj.emboj.7600486.

[18] Xiao ZX, Chen J, Levine AJ, Modjtahedi N, Xing J, Sellers WR, et al. Interaction between the retinoblastoma protein and the oncoprotein MDM2. Nature. 1995;375(6533):694–698. doi:10.1038/375694a0.

[19] Barak Y, Juven T, Haffner R, Oren M. mdm2 expression is induced by wild type p53 activity. The EMBO Journal. 1993;12.

[20] Duli V, Kaufmann WK, Wilson SJ, Tisty TD, Lees E, Harper JW, et al. p53-dependent inhibition of cyclin-dependent kinase activities in human fibroblasts during radiation-induced G1 arrest. Cell. 1994;76(6):1013–1023. doi:http://dx.doi.org/10.1016/0092-8674(94)90379-4.

[21] El-Deiry WS, Harper JW, O'Connor PM, Velculescu VE, Canman CE, Jackman J, et al. WAF1/CIP1 Is Induced in p53-mediated G1 Arrest and Apoptosis. Cancer Research. 1994;54(5):1169–1174.

[22] Fiscella M, Zhang H, Fan S, Sakaguchi K, Shen S, Mercer WE, et al. Wip1, a novel human protein phosphatase that is induced in response to ionizing radiation in a p53-dependent manner. Proceedings of the National Academy of Sciences. 1997;94(12):6048–6053.

[23] Suzuki HI, Yamagata K, Sugimoto K, Iwamoto T, Kato S, Miyazono K. Modulation of microRNA processing by p53. Nature. 2009;460(7254):529–533. doi:10.1038/nature08199.

[24] Polager S, Ginsberg D. p53 and E2f: partners in life and death. Nat Rev Cancer. 2009;9(10):738–748. doi:10.1038/nrc2718.

[25] Berkovich E, Ginsberg D. ATM is a target for positive regulation by E2F-1. Oncogene. 2003;22(2):161–167. doi:10.1038/sj.onc.1206144.

[26] Bueno MJ, Malumbres M. MicroRNAs and the cell cycle. Biochimica et Biophysica Acta (BBA) - Molecular Basis of Disease. 2011;1812(5):592–601. doi:https://doi.org/10.1016/j.bbadis.2011.02.002.

[27] Harper JW, Elledge SJ, Keyomarsi K, Dynlacht B, Tsai LH, Zhang P, et al. Inhibition of cyclin-dependent kinases by p21. Molecular Biology of the Cell. 1995;6(4):387–400. doi:10.1091/mbc.6.4.387.

[28] Chellappan SP, Hiebert S, Mudryj M, Horowitz JM, Nevins JR. The E2F transcription factor is a cellular target for the RB protein. Cell. 1991;65(6):1053–1061. doi:http://dx.doi.org/10.1016/0092-8674(91)90557-F.

[29] Hsieh JK, Chan FSG, OConnor DJ, Mittnacht S, Zhong S, Lu X. RB Regulates the Stability and the Apoptotic Function of p53 via MDM2. Molecular Cell. 1999;3(2):181–193. doi:http://dx.doi.org/10.1016/S1097-2765(00)80309-3.

[30] Liu Q, Fu H, Sun F, Zhang H, Tie Y, Zhu J, et al. miR-16 family induces cell cycle arrest by regulating multiple cell cycle genes. Nucleic Acids Research. 2008;36(16):5391–5404. doi:10.1093/nar/gkn522.

[31] Zhang X, Wan G, Mlotshwa S, Vance V, Berger FG, Chen H, et al. Oncogenic Wip1 Phosphatase Is Inhibited by miR-16 in the DNA Damage Signaling Pathway. Cancer Research. 2010;70(18):7176–7186. doi:10.1158/0008-5472.CAN-10-0697.

[32] Donzelli M, Draetta GF. Regulating mammalian checkpoints through Cdc25 inactivation. EMBO reports. 2003;4(7):671–677. doi:10.1038/sj.embor.embor887.

[33] Takaki T, Fukasawa K, Suzuki-Takahashi I, Hirai H. Cdk-mediated phosphorylation of pRB regulates HDAC binding in vitro. Biochemical and Biophysical Research Communications. 2004;316(1):252–255. doi:http://dx.doi.org/10.1016/j.bbrc.2004.02.044.

[34] Spring L, Bardia A, Modi S. Targeting the cyclin D-cyclin-dependent kinase (CDK)4/6-retinoblastoma pathway with selective CDK 4/6 inhibitors in hormone receptor-positive breast cancer: rationale, current status, and future directions. Discovery medicine. 2016;21(113):65–74.
