# Supplementary material for: MicroRNA-16 feedback loop with p53 and Wip1 can regulate cell fate determination between apoptosis and senescence in DNA damage response
Source: PLoS One. 2017 Oct 2;12(10):e0185794. doi: 10.1371/journal.pone.0185794 (PMC5624635; doi:10.1371/journal.pone.0185794)
Supplement: S1 Table — Official name of molecules used in the model. (PDF) [file pone.0185794.s003.pdf]

# Molecular Definition of Network Elements

| Node | Description |
|---|---|
| S | DNA damage: 0 (no damage), (1) reparable and (2) irreparable damage |
| ATR | Ataxia telangiectasia and Rad3 related protein |
| ATM | Ataxia telangiectasia mutated protein |
| miR16 | microRNA 16-1 |
| p53 | Tumor supressor p53 protein |
| Mdm2 | E3 ubiquitin protein ligase homolog protein |
| Wip1 | Protein Phosphatase 1D |
| p21 | Cyclin-dependent kinase inhibitor 1A protein |
| pRB | Retinoblastoma 1 protein |
| E2F1 | E2F1 transcription factor 1 |
| Cdk2cE | Cyclin dependent kinase 2 / Cyclin E Complex |
| Cdc25 | Cell division cycle 25A protein |
| Cdk46cD | Cyclin dependent kinase 4 and 6 / Cyclin D Complex |
