# Supplementary material for: MicroRNA-16 feedback loop with p53 and Wip1 can regulate cell fate determination between apoptosis and senescence in DNA damage response
Source: PLoS One. 2017 Oct 2;12(10):e0185794. doi: 10.1371/journal.pone.0185794 (PMC5624635; doi:10.1371/journal.pone.0185794)
Supplement: S2 Table — List of GoF and LoF perturbations and corresponding phenotypes. (PDF) [file pone.0185794.s004.pdf]

PHENOTYPES ACCORDING TO NODE PERTURBATIONS

In silico perturbations consist in fixing a variable to a single value or to a range of values. For variables in the interval between 0 and 2, there are five possible perturbations, while boolean nodes can have only three possible perturbations, see below.

In our results, we associate stable phenotypes with stable states and cyclic attractors with transient phenotypes. Then, based on experimental evidence, the interpretation of phenotypes produced by the model is as follows: a proliferative state corresponds to a stable state where the G1/S phase transition promoters (E2F1,Cdk2cE, Cdk46cD) are activated, implying that the cell cycle was not arrested at the G1 phase. There are two types of cycle arrests: stable and transient ones. Stable arrests correspond to stable states where the phase transition promoters are inhibited, and transient arrests correspond to a cyclic attractor. Senescence corresponds to a stable arrest where in addition both pRB and p21 are activated.

In table, each line presents a possible perturbation in the second to the sixth column for a node in the first column. The seventh column presents the stable states found and, according to text above, the phenotypes are classified as: P (proliferation), S (senescence), A (apoptosis), and Ar (arrest) in the case of an incompletely characterized arrest. The values of the variables in each stable state are presented in the order of the list of variables in the seventh column. The existence of a transient arrest (a cyclic attractor), is indicated in the last column, where the value indicates the number of attractors found. The eighth column indicates, whether or not, a bistable behavior was found for the stable states in the seventh column.

Perturbations types: (1) K.O.: variable=0
(2) E[0,1]: variable=[0-1] (value of the variable maintained in the interval between 0 and 1)
(3) E1: variable=1
(4) E[1,2]: variable=[1-2] (value of the variable maintained in the interval between 1 and 2)
(5) E[2]: variable=2

Node	K.O.	E[0,1]	E1	E[1,2]	E2	Types of phenotypes: P (proliferation), S (senescence), A (apoptosis), Ar (arrest)<br>**S, ATM,ATR,miR16,p53,MDM2,Wip1,p21,pRB,E2F1,Cdk2cE,Cdc25,Cdk46cD**	Bistable state?	Number of cycles<br>**Trans. arrests**
ATM	X					0000000001111 (P)<br>2020101110010 (S)	No	1
		X				0000000001111 (P)<br>2020101110010 (S)	No	1
			X			2120101110000 (S)	No	2
				X		2120101110000 (S)<br>2222200110000 (A)	Yes	2
					X	0200101110010 (S)<br>0202200110010 (A)<br>1210101110000 (S)<br>1212200110000 (A)<br>2220101110000 (S)<br>2222200110000 (A)	Yes	0
ATR	X					0000000001111 (P)<br>1000000000111 (P)<br>2202200110010 (A)	No	0
		X				0000000001111 (P)<br>2212200110000 (A)	No	1
			X			2212200110000 (A)	No	2

Node	K.O.	E[0,1]	E1	E[1,2]	E2	Output		# Cycles
ATR				X		2020101110010 (S)<br>2222200110000 (A)	Yes	2
					X	0020101110010 (S)<br>1020101110010 (S)<br>2020101110010 (S)<br>2222200110000 (A)	Yes	0
p53	X					0000000001111 (P)<br>1110000001100 (Ar)<br>2220000010000 (Ar)	No	0
		X				0000000001111 (P)<br>2020101110010 (S)	No	1
			X			0000101110010 (S)<br>1010101110010 (S)<br>2020101110010 (S)	No	0
				X		0000101110010 (S)<br>1110101110010 (S)<br>2020101110010 (S)<br>2222200110000 (A)	Yes	0
					X	0002210000010 (A)<br>1012210000010 (A)<br>2222200110000 (A)	No	0
miR16	X					0000000001111 (P)<br>2020101110010 (S)	No	1
		X				0000000001111 (P)<br>2020101110010 (S)<br>2221200110000 (A)	Yes	1

Node	K.O.	E[0,1]	E1	E[1,2]	E2	Output		# Cycles
miR16			X			2221200110000 (A)	No	2
				X		2222200110000 (A)	No	2
					X	2222200110000 (A)	No	2
Mdm2	X					0000000001111 (P)<br>1012200110010 (A)<br>2020101110010 (S)<br>2222200110000 (A)	Yes	0
			X			0000010001111 (P)<br>1110010001100 (Ar)	No	1
Wip1	X					0000000001111 (P)<br>2222200110000 (A)	No	1
			X			0000001001111 (P)<br>1010001001111 (P)<br>2020101110010 (S)	No	0
p21	X					0000000001111 (P)<br>2020101000111 (P)<br>2222200010000 (A)	Yes	1
			X			2020101110010 (S)<br>2222200110000 (A)	Yes	1
pRB	X					0000000001111 (P)<br>2020101100010 (Ar)<br>2222200100000 (A)	Yes	1
			X			0000010010111 (P)<br>1010010010111 (P)<br>2020101110010 (S)<br>2222200110000 (A)	Yes	0

Node	K.O.	E[0,1]	E1	E[1,2]	E2	Output		# Cycles
E2F1	X					0000000000111 (P)<br>2020101110010 (S)<br>2222200110000 (A)	Yes	1
		X				0000000001111 (P)<br>2020101110010 (S)<br>2222200110000 (A)	Yes	1
			X			0000000001111 (P)<br>2222200111000 (A)	No	1
				X		0000000001111 (P)<br>2222200111000 (A)	No	1
					X	0202200112010 (A)<br>1212200112000 (A)<br>2222200112000 (A)	No	0
Cdk2cE	X					0000000001011 (P)<br>2020101110010 (S)<br>2222200110000 (A)	Yes	1
			X			0000000001111 (P)<br>2020101100110 (S)<br>2222200100100 (A)	Yes	1
Cdc25	X					0000000001100 (Ar)<br>2020101110000 (S)<br>2222200110000 (A)	Yes	1
			X			0000000001111 (P)<br>2020101110010 (S)<br>2222200110010 (A)	Yes	1
Cdk46cD	X					0000000001110 (P)<br>2020101110010 (S)<br>2222200110000 (A)	Yes	1

			X			0000000001111 (P)<br>2020101100011 (Ar)<br>2222200100001 (A)	Yes	1
